# The applicability of the new media platform “Urological Surgery Learning Notes” in continuing medical education for urologists

**DOI:** 10.1186/s12909-026-08853-0

**Published:** 2026-02-27

**Authors:** Shiying Tang, Peng Hong, Guodong Zhu, Wei Guo, Xun Zhao, Jiyuan Chen, Lei Liu, Hongxian Zhang, Chunlei Xiao, Ke Liu, Jian Lu, Lulin Ma, Kai Hong, Shudong Zhang, Zhuo Liu

**Affiliations:** 1 Department of Urology, Peking University Third Hospital, Beijing, 100191 China; 2 Department of Urology, Chinese PLA General Hospital Ninth Medical Center, Beijing, 100101 China; 3 Department of Urology, Yan’an Traditional Chinese Medical Hospital, Peking University Third Hospital Yan’an Branch), Yan’an, Shaanxi 716099 China

**Keywords:** Urology, Specialized Medical Education, New Media Platform, Online Education

## Abstract

**Objective:**

To explore the applicability of the new media platform “Urological Surgery Learning Notes” in the continuing education of urology specialists.

**Methods:**

In December 2024, 390 subjects were recruited. Educational content, questions, and interactive materials related to the diagnosis, treatment, and surgery of urological diseases were disseminated via the WeChat public platform “Urological Surgery Learning Notes.” Post-instruction surveys were conducted to assess knowledge acquisition, attitudes, and educational outcomes.

**Results:**

Among the 390 subjects, 313 (80.26%) used the platform’s educational resource library more than once per week. 270 participants (69.23%) showed greater interest in learning content related to surgical techniques and precautions, while 340 (87.18%) preferred the video tutorial format offered by the platform. Regarding comprehension of different urological teaching content, 84.6% participants found the resource library’s content understandable. 88.19% participants believed it was effective or highly effective in improving urological surgical skills or clinical decision-making abilities. However, only 11.3% participants reported applying content from this resource library to more than 20 cases in their clinical practice.

**Conclusion:**

The use of the WeChat public platform-based educational resource library for continuing education of urology specialists demonstrated high acceptance, recognition, and applicability, proving to be feasible. However, its application process requires further enhancement based on the actual case mix and clinical realities of different hospitals.

**Supplementary Information:**

The online version contains supplementary material available at 10.1186/s12909-026-08853-0.

## Introduction

Compared to undergraduate medical education, specialized medical education focuses more on clinical practice related to specific diseases within a specialty. Traditional urological surgical training is structured around residency programs, operating room apprenticeships, and continuing education activities such as conferences and workshops [1]. While essential, this model faces challenges including limited accessibility, scheduling inflexibility, and high costs, which can hinder continuous learning for practicing specialists [1]. Cultivating competent specialists is crucial for physicians’ subsequent development [2]. However, challenges remain in specialized medical education, such as relatively singular teaching models, lack of standardized teaching materials, and varying teaching abilities among instructors [3].

The integration of digital and social media platforms into medical education has emerged to address some of these gaps. Platforms like YouTube, Twitter, WhatsApp, and WeChat are increasingly used for knowledge dissemination, peer communication, and blended learning, demonstrating potential to enhance engagement and utilize fragmented time [4, 5]. Furthermore, it can present abstract and complex medical knowledge in a visualized manner through video explanations, graphic illustrations, etc., which is particularly effective in surgical teaching, greatly facilitating students’ understanding and absorption of knowledge points [6]. However, much of the existing application is either generic or fragmented, lacking a structured, curriculum-aligned design specifically for the continuing professional development of surgical specialists [7]. Furthermore, the near-ubiquitous adoption of WeChat among medical professionals in China offers a unique opportunity to deliver educational content within an already integrated ecosystem.

Consequently, the key question has evolved from whether social media can be used, to how it can be best structured to achieve specific, high-level educational outcomes in specialized training [8]. In response, our team developed the WeChat public platform “Urological Surgery Learning Notes” as a structured, disease-specific educational resource library, designed explicitly for the education of urologists. This study evaluates its real-world applicability, user experience, and perceived effectiveness, thereby examining the advantages, challenges, and potential optimizations of a dedicated new media platform in surgical specialist education.

## Methods

### Subjects

A convenience sampling strategy was employed to recruit participants from the existing user base of the “Urological Surgery Learning Notes” WeChat public platform. During December 2024, an online questionnaire link was distributed through the platform’s official channel and shared within several large urology professional WeChat groups in China to broaden reach. Participation was voluntary and anonymous. 390 urologists from hospitals across various tiers in China were selected as subjects. For the purpose of this study, the term “urology specialists” encompassed physicians at different stages of urological practice, including resident physicians in standardized training, fellows in sub-specialty training, and attending physicians who had completed their formal specialization. The key inclusion criterion was that all participants had actively used the “Learning Notes” WeChat public platform for learning related to the diagnosis, treatment, and surgery of urological diseases. 

### Characteristics of the “Urological Surgery Learning Notes” Platform

The “Urological Surgery Learning Notes” is a curated, expert-led WeChat public account designed as a structured digital resource library for urological education. It is not an open, user-generated content platform. All educational content (including articles, surgical videos, and graphic explanations) is created and published exclusively by the project’s editorial team, which consists of experienced urologists and medical educators. Content submission is by invitation only, and each piece undergoes internal review by at least two senior team members for clinical accuracy, educational relevance, and appropriateness before release. The platform does not allow public users to upload content or post open comments, thereby inherently minimizing the risk of unvetted, disorienting, or inappropriate material being disseminated.

### Research methods and subject management

The “Learning Notes” new media resource library was categorized by disease, including: adrenal and retroperitoneal tumor surgery, prostate cancer surgery, partial nephrectomy, radical nephrectomy, transurethral resection of bladder tumor (TURBT), radical cystectomy with urinary diversion, testicular and penile cancer surgery, benign prostatic hyperplasia (BPH) surgery, ureteral or urethral stricture surgery, ureteroscopic stone surgery, percutaneous nephrolithotomy (PCNL), renal transplantation, andrology, medical therapy for urological tumors, urological emergencies, and coordination of urological surgical care.

A structured questionnaire was designed using software to survey the subjects, covering: basic information (gender, hospital level, professional title, years working in urology), usage patterns (source of awareness, frequency of use, focus areas, learning format, clinical application, comprehension level, perceived improvement in diagnostic and therapeutic skills), and suggestions for improvement. The questionnaire was open for one week, after which data were exported for analysis of teaching effectiveness and feedback.

### Questionnaire development and testing

The structured questionnaire used in this study was developed specifically for this research and has not been published elsewhere. It was designed to evaluate the platform’s utility in continuing urological education. Initial items were generated based on the platform’s content categories and key learning outcomes (e.g., comprehension, perceived effectiveness, clinical application). The draft questionnaire was reviewed by three senior urologists for content relevance and clarity. It was then pilot-tested with 8 urologists who were familiar with the platform but not included in the final study sample. Feedback from the pilot phase was used to adjust item wording, simplify response scales, and ensure the questionnaire was understandable and comprehensive. The complete questionnaire (English translation) is available as Supplementary Table 1.

### Statistical analysis

All data management and statistical analyses were performed using SPSS version 22.0. Descriptive statistics were used to summarize the data. Continuous variables are presented as mean ± standard deviation (SD). Categorical variables are presented as counts and percentages (n, %).

## Results

### Basic information of subjects

All 390 distributed questionnaires were returned, resulting in a 100% response rate. The subjects’ ages ranged from 24 to 60 years, with an average of 36.14 ± 6.52 years. Regarding hospital level: 219 (56.15%) worked in tertiary Grade A hospitals, 83 (21.28%) in tertiary Grade B hospitals, and 88 (22.56%) in secondary hospitals. Professional titles: 109 (27.95%) held associate senior titles or above, 201 (51.54%) held intermediate titles, and 80 (20.51%) held junior titles. Experience in urology: 161 (41.28%) had over 10 years of experience, 96 (24.62%) had 5–10 years of experience.

### Usage patterns of the new media platform resource library

313 participants (80.26%) used the resource library more than once per week. Regarding focus areas: 270 (69.23%) paid more attention to content related to surgical techniques and precautions, while 245 (62.82%) focused more on detailed surgical steps. Regarding learning format: 340 (87.18%) preferred video tutorials, and 296 (75.9%) preferred detailed graphic explanations. The top three most followed teaching contents were BPH surgery (84.62%), kidney cancer surgery (81.03%), and prostate cancer surgery (80.00%).

### Comprehension and clinical application of the resource library content

Regarding comprehension: For different urological teaching contents, 84.6% participants found the content understandable or easy to understand. The highest comprehension rates were for ureteroscopic stone surgery (92.56%), TURBT (92.05%), and BPH surgery (91.54%). The lowest comprehension rate was for renal transplantation (62.56%). Details are shown in Table 1.

**Table 1:** Overall comprehension level of the content in “Learning Notes” among the 390 subjects

Item	Do not understand at all	Do not understand	Neutral	Understand	Easy to understand
Adrenal and Retroperitoneal Tumor Surgery	3(0.77%)	3(0.77%)	43(11.03%)	185(47.44%)	156(40%)
Prostate Cancer Surgery	4(1.03%)	4(1.03%)	41(10.51%)	194(49.74%)	147(37.69%)
Partial Nephrectomy	4(1.03%)	4(1.03%)	33(8.46%)	184(47.18%)	165(42.31%)
Radical Nephrectomy	5(1.28%)	4(1.03%)	33(8.46%)	177(45.38%)	171(43.85%)
Transurethral Resection of Bladder Tumor	1(0.26%)	3(0.77%)	27(6.92%)	166(42.56%)	193(49.49%)
Radical Cystectomy	6(1.54%)	6(1.54%)	55(14.1%)	187(47.95%)	136(34.87%)
Testicular and Penile Cancer Surgery	5(1.28%)	5(1.28%)	54(13.85%)	176(45.13%)	150(38.46%)
Benign Prostatic Hyperplasia Surgery	2(0.51%)	1(0.26%)	30(7.69%)	151(38.72%)	206(52.82%)
Ureteral or Urethral Stricture Surgery	6(1.54%)	5(1.28%)	50(12.82%)	186(47.69%)	143(36.67%)
Ureteroscopic Stone Surgery	4(1.03%)	2(0.51%)	23(5.9%)	160(41.03%)	201(51.54%)
Percutaneous Nephrolithotomy	4(1.03%)	4(1.03%)	39(10%)	168(43.08%)	175(44.87%)
Renal Transplantation	25(6.41%)	23(5.9%)	98(25.13%)	133(34.1%)	111(28.46%)
Andrology	4(1.03%)	5(1.28%)	58(14.87%)	168(43.08%)	155(39.74%)
Medical Therapy for Urological Tumors	9(2.31%)	5(1.28%)	75(19.23%)	166(42.56%)	135(34.62%)
Urological Emergency Diseases	3(0.77%)	3(0.77%)	48(12.31%)	175(44.87%)	161(41.28%)
Coordination of Urological Surgical Care	10(2.56%)	6(1.54%)	71(18.21%)	167(42.82%)	136(34.87%)

Regarding perceived effectiveness: 88.19% participants believed the resource library was effective or highly effective in improving urological surgical skills or clinical decision-making abilities. The highest effectiveness ratings were for BPH surgery (94.10%), ureteroscopic stone surgery (93.08%), and TURBT (92.82%). The lowest effectiveness rating was for renal transplantation (71.54%). Details are shown in Table 2.

**Table 2:** Perceived effectiveness of “Learning Notes” in improving urological surgical skills or clinical diagnostic and therapeutic abilities among the 390 subjects

Item	Ineffective	Slightly Effective	Neutral	Effective	Highly Effective
Adrenal and Retroperitoneal Tumor Surgery	3(0.77%)	4(1.03%)	30(7.69%)	175(44.87%)	178(45.64%)
Prostate Cancer Surgery	1(0.26%)	4(1.03%)	33(8.46%)	179(45.9%)	173(44.36%)
Partial Nephrectomy	0(0%)	5(1.28%)	31(7.95%)	177(45.38%)	177(45.38%)
Radical Nephrectomy	3(0.77%)	4(1.03%)	31(7.95%)	167(42.82%)	185(47.44%)
Transurethral Resection of Bladder Tumor	2(0.51%)	5(1.28%)	21(5.38%)	171(43.85%)	191(48.97%)
Radical Cystectomy	4(1.03%)	7(1.79%)	46(11.79%)	167(42.82%)	166(42.56%)
Testicular and Penile Cancer Surgery	2(0.51%)	6(1.54%)	45(11.54%)	165(42.31%)	172(44.1%)
Benign Prostatic Hyperplasia Surgery	0(0%)	3(0.77%)	20(5.13%)	168(43.08%)	199(51.03%)
Ureteral or Urethral Stricture Surgery	4(1.03%)	6(1.54%)	37(9.49%)	185(47.44%)	158(40.51%)
Ureteroscopic Stone Surgery	1(0.26%)	5(1.28%)	21(5.38%)	166(42.56%)	197(50.51%)
Percutaneous Nephrolithotomy	1(0.26%)	8(2.05%)	29(7.44%)	172(44.1%)	180(46.15%)
Renal Transplantation	24(6.15%)	10(2.56%)	77(19.74%)	143(36.67%)	136(34.87%)
Andrology	2(0.51%)	7(1.79%)	43(11.03%)	167(42.82%)	171(43.85%)
Medical Therapy for Urological Tumors	4(1.03%)	7(1.79%)	41(10.51%)	172(44.1%)	166(42.56%)
Urological Emergency Diseases	1(0.26%)	7(1.79%)	33(8.46%)	174(44.62%)	175(44.87%)
Coordination of Urological Surgical Care	9(2.31%)	9(2.31%)	41(10.51%)	173(44.36%)	158(40.51%)

Regarding clinical application: 22.0% participants reported applying content from the resource library to more than 10 cases in their clinical practice, but only 11.3% applied it to more than 20 cases. The procedures with the highest application rates (> 30 cases) were ureteroscopic stone surgery (93 participants), BPH surgery (57 participants), PCNL (46 participants), and TURBT (33 participants). The lowest application rate was for renal transplantation. Details are shown in Table 3.

**Table 3:** Application of content from “Learning Notes” in the clinical practice of the 390 subjects

Item	< 10 Cases	> 10 Cases
Adrenal and Retroperitoneal Tumor Surgery	346(88.72%)	44(11.28%)
Prostate Cancer Surgery	330(84.62%)	60(15.38%)
Partial Nephrectomy	320(82.05%)	70(17.95%)
Radical Nephrectomy	309(79.23%)	81(20.77%)
Transurethral Resection of Bladder Tumor	268(68.72%)	122(31.28%)
Radical Cystectomy	358(91.79%)	32(8.21%)
Testicular and Penile Cancer Surgery	358(91.79%)	32(8.21%)
Benign Prostatic Hyperplasia Surgery	214(54.87%)	176(45.13%)
Ureteral or Urethral Stricture Surgery	335(85.9%)	55(14.1%)
Ureteroscopic Stone Surgery	193(49.49%)	197(50.51%)
Percutaneous Nephrolithotomy	257(65.9%)	133(34.1%)
Renal Transplantation	377(96.67%)	13(3.33%)
Andrology	288(73.85%)	102(26.15%)
Medical Therapy for Urological Tumors	309(79.23%)	81(20.77%)
Urological Emergency Diseases	299(76.67%)	91(23.33%)
Coordination of Urological Surgical Care	309(79.23%)	81(20.77%)

### Main difficulties or suggestions reported by subjects

After using the resource library, 122 participants suggested increasing the amount of surgical video content, 63 hoped for added instructor commentary and personal experience sharing within the videos, and 19 suggested enhancing interactive features, such as improving online discussion and Q&A functions.

## Discussion

Medical education plays a vital role in fostering clinical specialization, ensuring the improvement and development of medical standards, and promoting lifelong learning among all physicians [9, 10]. Urological medical education is particularly significant for both the standardized training of resident physicians and the continuing education of urology specialists [11, 12]. Current primary forms of urological specialized education include academic activities (international conferences, case discussions, etc.), online learning (continuing education platforms, etc.), practical training (simulation center exercises, etc.), and self-directed learning (reading medical journals/books, etc.). Surgical teaching is a crucial and practical component of continuing education for urologists. Traditional urological surgical teaching methods include textbook-based learning, video teaching, surgical observation, virtual surgery simulators, animal lab training, and clinical surgical training [13, 14]. However, traditional methods are often constrained by time, scheduling, facility availability, cost, and travel. New media resource libraries can serve as carriers for integrating and disseminating online medical educational resources, providing safer, more reliable, convenient, and efficient pathways for medical education [15–17].

Utilizing a WeChat public platform like “Learning Notes” to cultivate urological surgical thinking and skills represents a novel teaching method. Research underscores that visual content, such as images and videos, is crucial for effective dissemination on social media, with highly visual platforms being particularly popular for medical education [18]. “Learning Notes” directly addresses this by offering a wealth of surgical videos and graphic explanations. Furthermore, it overcomes a common social media drawback—the time-consuming nature of searching for relevant information [19]—through its structured, disease-categorized repository and efficient keyword search function. Its online learning functionality allows for flexible scheduling, enabling learners to consolidate and expand their knowledge anytime. Compared to traditional methods and fragmented general social media, “Learning Notes” offers distinct advantages such as ease of retrieval, high learning efficiency (utilizing fragmented time), and cost-effectiveness (all articles are free). Since its launch on December 31, 2021, the platform has published over 230 articles, broadly covering urological diseases like kidney cancer surgery, adrenal and retroperitoneal tumors, and lower urinary tract surgery.

In the era of networked new media, the integration of advanced information technology with teaching has spurred new trends in educational reform. Remote teaching systems break spatial barriers, reducing manpower costs and saving significant time [2]. A research by professor Tang on new media platforms in andrology continuing education showed that 66.67% of subjects felt no significant increase in learning pressure with online learning, highlighting its acceptability, and 80.25% expressed willingness to continue using this mode, indicating high recognition and acceptance of new media combined with online PBL [11]. The “Learning Notes” platform holds significant importance and promise in the surgical education of urology specialists, fostering self-directed learning, encouraging the summarization of classic clinical cases, and applying acquired knowledge and skills to clinical practice. It represents a more efficient, standardized, and distinctive new medical education model that can comprehensively enhance the diagnostic, therapeutic, and surgical skills of specialists and has promotional value.

Our study data indicate a strong preference for video tutorials (87.18%) and graphic explanations (75.90%), reflecting high recognition for multimedia learning resources. Furthermore, participants generally found the resource library content understandable, particularly for renal and prostate surgeries. The high frequency of use (80.26% used it more than once weekly) demonstrates its high acceptance and the frequent recourse to this resource for solving clinical problems. A majority of participants believed the resource library significantly improved their surgical skills or clinical decision-making abilities, with over 80% rating it as “effective” or “highly effective” for procedures like adrenal/retroperitoneal tumor surgery, prostate cancer surgery, and BPH surgery.

However, the study also revealed that most participants reported relatively low application of the learned content in their clinical practice. For instance, over half reported applying the knowledge to fewer than 5 cases for adrenal and retroperitoneal tumors, whereas procedures like TURBT, BPH surgery, ureteroscopic stone surgery, PCNL, and andrology saw more than 100 participants (> 25.6%) applying the knowledge to over 10 cases. The primary reason appears to be the relatively recent establishment of the platform, meaning content depth and breadth are still evolving. Additionally, the survey identified that the primary users are physicians from primary-level hospitals, where patient volumes for certain specific diseases might be lower, and the types of surgeries performed are limited. Therefore, enriching the platform’s resource library to comprehensively meet the diverse needs of hospitals at different levels is urgently needed.

This study has several limitations. First, the findings rely entirely on self-reported data, which are subject to biases such as social desirability and recall inaccuracy, and may not fully reflect objective measures of knowledge or skill improvement. Second, the questionnaire was newly developed for this platform; although it underwent expert review and pilot testing, its reliability and validity have not been formally established through standardized psychometric evaluation, which may affect the generalizability of the results. Third, as a feasibility and acceptability study, our sample consisted specifically of current platform users. While this provides valuable insights into the user experience, the positive perceptions reported here may not extend to urologists who are non-users or less receptive to digital education tools, which limits the generalizability of the findings to that broader population. Future research should incorporate objective assessments—such as knowledge tests, procedural outcome analysis, or independent skill evaluations—to complement self-reported findings. Nevertheless, the high response rate and detailed feedback provide a meaningful preliminary evaluation of the platform’s acceptability and perceived utility in continuing urological education.

In conclusion, the “Urological Surgery Learning Notes” WeChat public platform demonstrates high acceptance, recognition, and applicability for the continuing education of urology specialists and is feasible. However, its application process requires further refinement based on the actual patient case mix in different hospitals to strengthen its integration with clinical practice.

## Supplementary Information

Supplementary Material 1.

## Data Availability

The datasets used and analysed during the current study are available from the corresponding author on reasonable request.

## Abbreviations

TURBT: Transurethral resection of bladder tumor
BPH: Benign prostatic hyperplasia
PCNL: Percutaneous nephrolithotomy
