# Supplementary material for: The applicability of the new media platform “Urological Surgery Learning Notes” in continuing medical education for urologists
Source: BMC Med Educ. 2026 Feb 27;26:546. doi: 10.1186/s12909-026-08853-0 (PMC13041337; doi:10.1186/s12909-026-08853-0)
Supplement: Supplementary file 1 — Supplementary Material 1. [file 12909_2026_8853_MOESM1_ESM.docx]

**Questionnaire: Evaluation of the “Urological Surgery Learning Notes” WeChat Platform in Continuing Medical Education**

**Dear Urologist,**

This survey is conducted as part of a continuing medical education quality improvement initiative within the Department of Urology, Peking University Third Hospital, and is carried out in accordance with the Declaration of Helsinki. Your feedback is invaluable and will directly help us improve the quality and effectiveness of our educational resources. Please complete this questionnaire voluntarily, honestly, and objectively based on your actual experience. This study is anonymous, and no personally identifiable information will be collected. Submission of the completed questionnaire will be regarded as implied informed consent. Thank you very much for your cooperation and support!

**I. Demographic Information**

**1. Gender:**

- Male
- Female

**2. Level of your hospital:**

- Tertiary Grade A
- Tertiary Grade B
- Secondary Grade A
- Secondary Grade B
- Primary hospital

**3. Your professional title:**

- Chief Physician
- Associate Chief Physician
- Attending Physician
- Resident Physician

**4. Years of experience in urology:**

- < 1 year
- 1–3 years
- 3–5 years
- 5–10 years
- 10 years

**II. Usage Patterns and Evaluation**

**5. How did you first learn about the “Urological Surgery Learning Notes” educational resource library?** (Select up to 2)

- Hospital internal notice
- Colleague recommendation
- Academic conference
- Internet search
- Other (please specify): __________

**6. How often do you use this educational resource library per week?**

- Daily
- 3–5 times per week
- 1–2 times per week
- Occasionally
- Never

**7. What types of content do you primarily focus on when using this resource library?** (Select up to 2)

- Surgical videos
- Detailed surgical steps
- Surgical techniques and precautions
- Management of postoperative complications
- Relevant theoretical knowledge
- Other (please specify): __________

**8. What formats of learning resources do you prefer for urological surgery education?** (Select up to 2)

- Video tutorials
- Illustrated step-by-step explanations
- Interactive simulation software
- Live surgical demonstrations
- Offline workshops/seminars
- Other (please specify): __________

**9. Which urological disease/surgery topics do you most frequently access?** (Select all that apply)

- Kidney cancer surgery
- Upper tract urothelial carcinoma (renal pelvis, ureter)
- Bladder cancer surgery
- Prostate cancer surgery

**10. Which additional topics do you frequently access?** (Select all that apply)

- Adrenal and retroperitoneal tumors
- Renal transplantation
- Benign prostatic hyperplasia (BPH) surgery
- Stone surgery
- Andrology surgery

**For each of the following procedures, please estimate the number of patients in your clinical practice to whom you have applied knowledge or skills learned from the “Learning Notes” resource library:**

**11. Adrenal and retroperitoneal tumor surgery**

- < 5 cases
- 5–10 cases
- 10–20 cases
- 20–30 cases
- 30 cases

**12. Prostate cancer surgery**

- < 5 cases
- 5–10 cases
- 10–20 cases
- 20–30 cases
- 30 cases

**13. Partial nephrectomy**

- < 5 cases
- 5–10 cases
- 10–20 cases
- 20–30 cases
- 30 cases

**14. Radical nephrectomy**

- < 5 cases
- 5–10 cases
- 10–20 cases
- 20–30 cases
- 30 cases

**15. Transurethral resection of bladder tumor (TURBT)**

- < 5 cases
- 5–10 cases
- 10–20 cases
- 20–30 cases
- 30 cases

**16. Radical cystectomy with urinary diversion**

- < 5 cases
- 5–10 cases
- 10–20 cases
- 20–30 cases
- 30 cases

**17. Testicular and penile cancer surgery**

- < 5 cases
- 5–10 cases
- 10–20 cases
- 20–30 cases
- 30 cases

**18. Benign prostatic hyperplasia (BPH) surgery**

- < 5 cases
- 5–10 cases
- 10–20 cases
- 20–30 cases
- 30 cases

**19. Ureteral or urethral stricture surgery**

- < 5 cases
- 5–10 cases
- 10–20 cases
- 20–30 cases
- 30 cases

**20. Ureteroscopic stone surgery (rigid or flexible ureteroscopy)**

- < 5 cases
- 5–10 cases
- 10–20 cases
- 20–30 cases
- 30 cases

**21. Percutaneous nephrolithotomy (PCNL)**

- < 5 cases
- 5–10 cases
- 10–20 cases
- 20–30 cases
- 30 cases

**22. Renal transplantation**

- < 5 cases
- 5–10 cases
- 10–20 cases
- 20–30 cases
- 30 cases

**23. Andrology procedures** (e.g., varicocelectomy, hydrocelectomy, obstructive azoospermia, erectile dysfunction, concealed penis, cryptorchidism, reconstructive surgery)

- < 5 cases
- 5–10 cases
- 10–20 cases
- 20–30 cases
- 30 cases

**24. Medical therapy for urological tumors** (targeted therapy, immunotherapy, chemotherapy)

- < 5 cases
- 5–10 cases
- 10–20 cases
- 20–30 cases
- 30 cases

**25. Urological emergencies** (e.g., cardiopulmonary resuscitation, hemorrhage, urinary retention, infection, trauma, bladder foreign body)

- < 5 cases
- 5–10 cases
- 10–20 cases
- 20–30 cases
- 30 cases

**26. Coordination of urological surgical care** (e.g., robotic trocar placement, robotic suturing techniques, surgical sutures, patient positioning)

- < 5 cases
- 5–10 cases
- 10–20 cases
- 20–30 cases
- 30 cases

**27. Overall, how well do you understand the content of this educational resource library?**

- Very easy to understand
- Understandable
- Neutral
- Difficult to understand
- Cannot understand at all

**28. Overall, how effective do you believe this resource library is in improving your urological surgical skills?**

- Highly effective
- Effective
- Neutral
- Slightly effective
- Ineffective

**29. What are the main difficulties or problems you have encountered when using this resource library?** (Select all that apply)

- Infrequent content updates
- Insufficient content coverage
- Low video quality
- User-unfriendly interface
- Lack of interaction and Q&A features
- Other (please specify): __________

**30. Do you frequently access learning resources via mobile devices (smartphone or tablet)? How would you suggest improving the mobile user experience?**

- Frequently use; hope to optimize interface layout for small screens
- Frequently use; hope to add offline download function
- Occasionally use; no specific needs
- Rarely use

**III. Suggestions for Improvement**

**31. In your opinion, what aspects of the “Urological Surgery Learning Notes” new media educational resource library could be further improved or expanded? Please briefly describe:**

**End of Questionnaire**
**Thank you for your valuable feedback!**
